# Data from quantitative serum proteomic analysis after laparoscopic gastric plication

**DOI:** 10.1016/j.dib.2019.104077

**Published:** 2019-05-30

**Authors:** Parisa Savedoroudi, Tue Bjerg Bennike, Kenneth Kastaniegaard, Mohammad Talebpour, Alireza Ghassempour, Allan Stensballe

**Affiliations:** a Medicinal Plants and Drugs Research Institute, Shahid Beheshti University, Tehran, Iran; b Department of Health Science and Technology, Aalborg University, Denmark; c Laparoscopic Surgery Ward, Sina Hospital, Tehran University of Medical Sciences, Tehran, Iran

**Keywords:** Bariatric surgery, Laparoscopic gastric plication, Obesity, Proteomics

## Abstract

Bariatric surgery is an effective treatment for morbid obesity with a sustained weight loss and improvements in metabolic syndrome. We present a label free quantitative shotgun proteomics approach to analyze the serum proteome of obese people who underwent Laparoscopic Gastric Plication (LGP) as a new bariatric surgery. Pre-surgery serum samples of obese individuals were compared with the serum of the same subjects 1–2 months post-surgery (T1) and 4–5 months post-surgery (T2). The data provide a list of 224 quantifiable proteins with at least two unique peptides that were quantifiable in at least 70% of samples. Gene ontology biological processes and molecular functions of differentially regulated proteins between pre- and post-surgery samples were investigated using WebGestalt online tool. In addition, molecular networks of differentially abundant proteins were determined through Ingenuity Pathway Analysis (IPA) software. This report is related to the research article entitled “Serum proteome changes and accelerated reduction of fat mass after Laparoscopic Gastric Plication in morbidly obese patients” (Savedoroudi et al. [1]). Proteomics data have been deposited to the ProteomeXchange Consortium (http://proteomecentral.proteomexchange.org) via the PRIDE partner repository through the identifier PXD010528.

**Table:** Specifications Table

Subject area	Biology
More specific subject area	Serum proteomic analysis of obese people undergoing Laparoscopic gastric plication as a relatively new bariatric surgical technique.
Type of data	Raw data, Tables and excel file
How data was acquired	Data was acquired by LC-MS/MS using a UPLC-nanoESI MS/MS setup with a NanoRSLC system (Dionex, CA, USA) coupled to a Q Exactive HF mass spectrometer (Thermo Scientific, Waltham, USA). The data was analyzed with MaxQuant (version 1.6.0.1).
Data format	Raw and analyzed data
Experimental factors	Human serum samples were analyzed pre-surgery and post-surgery. Initially, they were depleted of the six most abundant serum proteins.
Experimental features	Label-free quantitative proteomics approach was used to analyze the serum samples. Proteins were digested using filter aided sample preparation. LC-MS/MS was used to analyze the purified peptides. The raw-files were analyzed with MaxQuant software (version 1.6.0.1) against the Uniprot human reference FASTA database (August 2017) and processed with Perseus version 1.6.0.2. Biological process and molecular function of differentially regulated proteins were determined using WebGestalt online tool. Meanwhile, their molecular networks were identified by IPA software.
Data source location	Laboratory of Medical Mass Spectrometry, Department of Health Science and Technology, Aalborg University, Denmark.
Data accessibility	Data are available within this article and from ProteomeXchange Consortium (http://proteomecentral.proteomexchange.org) via the PRIDE partner repository with the dataset identifier PXD010528.

**Table:** 

**Value of the data** • These data reveal the effect of laparoscopic gastric plication (LGP) on the proteome profile of obese people for the first time. • Data in this article provide information about the biological processes, molecular functions and molecular networks of differentially regulated proteins between pre- and post-surgery subjects. • These findings provide new insight into the underlying molecular mechanism associated with laparoscopic gastric plication that could be useful for further study in development of non-surgical weight loss strategies.

## 1 Data

This report is associated with the research article aimed at investigating the effect of weight loss due to laparoscopic gastric plication (LGP) as a new bariatric surgical procedure on the human serum proteome [1]. A total of 288 proteins was identified using a shotgun label-free proteomics experiment, of which 224 proteins were quantifiable with at least two unique peptides in 70% of samples or more (Supplementary Table 1). The raw mass data have been deposited to the ProteomeXchange Consortium (http://proteomecentral.proteomexchange.org) via the PRIDE partner repository [2] with the dataset identifier PXD010528. The list of submitted proteomics raw-files into the ProteomeXchange and corresponding sample names are shown in Table 1. Significantly regulated proteins between pre- and post-surgery samples were discussed in detail in Savedoroudi et al. [1]. Gene ontology enrichment analysis for biological process and molecular function of differentially regulated proteins at T1 and T2 are represented in Table 2 and Table 3, respectively. In Table 4, molecular networks of differentially regulated proteins are shown.

**Table 1:** Description of file-names and MaxQuant output in the ProteomeXchange repository PXD010528. MS files were analyzed in MaxQuant. All samples were analyzed in triplicates. Timepoint 1: 1–2 months post-surgery; Timepoint 2: 4–5 months post-surgery.

Raw file	Sample	MS system
2bef-1	2/Before surgery	Q Exactive HF
2bef-2	2/Before surgery	Q Exactive HF
2bef-3	2/Before surgery	Q Exactive HF
2aft1-1	2/After surgery at timepoint 1	Q Exactive HF
2aft1-2	2/After surgery at timepoint 1	Q Exactive HF
2aft1-3	2/After surgery at timepoint 1	Q Exactive HF
3bef-1	3/Before surgery	Q Exactive HF
3bef-2	3/Before surgery	Q Exactive HF
3bef-3	3/Before surgery	Q Exactive HF
3aft1-1	3/After surgery at timepoint 1	Q Exactive HF
3aft1-2	3/After surgery at timepoint 1	Q Exactive HF
3aft1-3_170725101835	3/After surgery at timepoint 1	Q Exactive HF
3aft2-1	3/After surgery at timepoint 2	Q Exactive HF
3aft2-2	3/After surgery at timepoint 2	Q Exactive HF
3aft2-3	3/After surgery at timepoint 2	Q Exactive HF
4bef-1	4/Before surgery	Q Exactive HF
4bef-2	4/Before surgery	Q Exactive HF
4bef-3	4/Before surgery	Q Exactive HF
4aft1-1	4/After surgery at timepoint 1	Q Exactive HF
4aft1-2	4/After surgery at timepoint 1	Q Exactive HF
4aft1-3	4/After surgery at timepoint 1	Q Exactive HF
5bef-1	5/Before surgery	Q Exactive HF
5bef-2	5/Before surgery	Q Exactive HF
5bef-3	5/Before surgery	Q Exactive HF
5aft1-1	5/After surgery at timepoint 1	Q Exactive HF
5aft1-2	5/After surgery at timepoint 1	Q Exactive HF
5aft1-3	5/After surgery at timepoint 1	Q Exactive HF
5aft2-1	5/After surgery at timepoint 2	Q Exactive HF
5aft2-2	5/After surgery at timepoint 2	Q Exactive HF
5aft2-3	5/After surgery at timepoint 2	Q Exactive HF
6bef-1	6/Before surgery	Q Exactive HF
6bef-2	6/Before surgery	Q Exactive HF
6bef-3	6/Before surgery	Q Exactive HF
6aft1-1	6/After surgery at timepoint 1	Q Exactive HF
6aft1-2	6/After surgery at timepoint 1	Q Exactive HF
6aft1-3	6/After surgery at timepoint 1	Q Exactive HF
6aft2-1	6/After surgery at timepoint 2	Q Exactive HF
6aft2-2	6/After surgery at timepoint 2	Q Exactive HF
6aft2-3	6/After surgery at timepoint 2	Q Exactive HF
7bef-1	7/Before surgery	Q Exactive HF
7bef-2	7/Before surgery	Q Exactive HF
7bef-3	7/Before surgery	Q Exactive HF
7aft1-1	7/After surgery at timepoint 1	Q Exactive HF
7aft1-2	7/After surgery at timepoint 1	Q Exactive HF
7aft1-3	7/After surgery at timepoint 1	Q Exactive HF
7aft2-1	7/After surgery at timepoint 2	Q Exactive HF
7aft2-2	7/After surgery at timepoint 2	Q Exactive HF
7aft2-3	7/After surgery at timepoint 2	Q Exactive HF
8bef-1	8/Before surgery	Q Exactive HF
8bef-2	8/Before surgery	Q Exactive HF
8bef-3	8/Before surgery	Q Exactive HF
8aft1-1	8/After surgery at timepoint 1	Q Exactive HF
8aft1-2	8/After surgery at timepoint 1	Q Exactive HF
8aft1-3_170725085836	8/After surgery at timepoint 1	Q Exactive HF
8aft2-1	8/After surgery at timepoint 2	Q Exactive HF
8aft2-2	8/After surgery at timepoint 2	Q Exactive HF
8aft2-3	8/After surgery at timepoint 2	Q Exactive HF
9bef-1	9/Before surgery	Q Exactive HF
9bef-2	9/Before surgery	Q Exactive HF
9bef-3	9/Before surgery	Q Exactive HF
9aft1-1	9/After surgery at timepoint 1	Q Exactive HF
9aft1-2	9/After surgery at timepoint 1	Q Exactive HF
9aft1-3	9/After surgery at timepoint 1	Q Exactive HF
10bef-1	10/Before surgery	Q Exactive HF
10bef-2	10/Before surgery	Q Exactive HF
10bef-3	10/Before surgery	Q Exactive HF
10aft1-1	10/After surgery at timepoint 1	Q Exactive HF
10aft1-2	10/After surgery at timepoint 1	Q Exactive HF
10aft1-3	10/After surgery at timepoint 1	Q Exactive HF
10aft2-1	10/After surgery at timepoint 2	Q Exactive HF
10aft2-2	10/After surgery at timepoint 2	Q Exactive HF
10aft2-3	10/After surgery at timepoint 2	Q Exactive HF
11bef-1	11/Before surgery	Q Exactive HF
11bef-2	11/Before surgery	Q Exactive HF
11bef-3	11/Before surgery	Q Exactive HF
11aft1-1	11/After surgery at timepoint 1	Q Exactive HF
11aft1-2	11/After surgery at timepoint 1	Q Exactive HF
11aft1-3	11/After surgery at timepoint 1	Q Exactive HF
11aft2-1	11/After surgery at timepoint 2	Q Exactive HF
11aft2-2	11/After surgery at timepoint 2	Q Exactive HF
11aft2-3	11/After surgery at timepoint 2	Q Exactive HF
12bef-1	12/Before surgery	Q Exactive HF
12bef-2	12/Before surgery	Q Exactive HF
12bef-3	12/Before surgery	Q Exactive HF
12aft1-1	12/After surgery at timepoint 1	Q Exactive HF
12aft1-2	12/After surgery at timepoint 1	Q Exactive HF
12aft1-3	12/After surgery at timepoint 1	Q Exactive HF
14bef-1	14/Before surgery	Q Exactive HF
14bef-2	14/Before surgery	Q Exactive HF
14bef-3	14/Before surgery	Q Exactive HF
14aft1-1	14/After surgery at timepoint 1	Q Exactive HF
14aft1-2	14/After surgery at timepoint 1	Q Exactive HF
14aft1-3	14/After surgery at timepoint 1	Q Exactive HF
14aft2-1	14/After surgery at timepoint 2	Q Exactive HF
14aft2-2	14/After surgery at timepoint 2	Q Exactive HF
14aft2-3	14/After surgery at timepoint 2	Q Exactive HF
15bef-1	15/Before surgery	Q Exactive HF
15bef-2	15/Before surgery	Q Exactive HF
15bef-3	15/Before surgery	Q Exactive HF
15aft1-1	15/After surgery at timepoint 1	Q Exactive HF
15aft1-2	15/After surgery at timepoint 1	Q Exactive HF
15aft1-3	15/After surgery at timepoint 1	Q Exactive HF
17bef-1	17/Before surgery	Q Exactive HF
17bef-2	17/Before surgery	Q Exactive HF
17bef-3	17/Before surgery	Q Exactive HF
17aft1-1	17/After surgery at timepoint 1	Q Exactive HF
17aft1-2	17/After surgery at timepoint 1	Q Exactive HF
17aft1-3	17/After surgery at timepoint 1	Q Exactive HF
17aft2-1	17/After surgery at timepoint 2	Q Exactive HF
17aft2-2	17/After surgery at timepoint 2	Q Exactive HF
17aft2-3	17/After surgery at timepoint 2	Q Exactive HF
18bef-1	18/Before surgery	Q Exactive HF
18bef-2	18/Before surgery	Q Exactive HF
18bef-3	18/Before surgery	Q Exactive HF
18aft1-1	18/After surgery at timepoint 1	Q Exactive HF
18aft1-2	18/After surgery at timepoint 1	Q Exactive HF
18aft1-3	18/After surgery at timepoint 1	Q Exactive HF
21bef-1	21/Before surgery	Q Exactive HF
21bef-2	21/Before surgery	Q Exactive HF
21bef-3	21/Before surgery	Q Exactive HF
21aft1-1	21/After surgery at timepoint 1	Q Exactive HF
21aft1-2	21/After surgery at timepoint 1	Q Exactive HF
21aft1-3	21/After surgery at timepoint 1	Q Exactive HF
**The MaxQuant output in folder "txt" contains a range of files containing important search information. Below file was used for further processing in Perseus post-analysis program and quantitative analysis.**		
proteinGroups.txt	File containing all proteins with corresponding label free quantitative information

**Table 2:** Enrichment analysis of Gene ontology biological process and molecular function for differentially abundant proteins at T1, using WebGestalt online tool.

Gen set	Description	P-value	Overlap Gen-ID
Biological Process			
GO:0051223	regulation of protein transport	4.87E-05	CRP, GPLD1, APOA1, APOA2, APOD, IL1RAP, LCP1, SRGN, RBP4, SAA1, CD14, ADIPOQ
GO:0070201	regulation of establishment of protein localization	8.84E-05	CRP, GPLD1, APOA1, APOA2, APOD, IL1RAP, LCP1, SRGN, RBP4, SAA1, CD14, ADIPOQ
GO:0034284	response to monosaccharide	0.000109	GPLD1, APOA2, SERPINF1, APOM, SPARC, THBS1, ADIPOQ
GO:0032880	regulation of protein localization	0.000256	CRP, GPLD1, APOA1, APOA2, APOD, IL1RAP, LCP1, SRGN, RBP4, SAA1, CD14, ADIPOQ
GO:0009743	response to carbohydrate	0.000315	GPLD1, APOA2, SERPINF1, APOM, SPARC, THBS1, ADIPOQ
GO:0009746	response to hexose	0.000532	GPLD1, APOA2, SERPINF1, APOM, THBS1, ADIPOQ
GO:0009749	response to glucose	0.000532	GPLD1, APOA2, SERPINF1, APOM, THBS1, ADIPOQ
GO:0050707	regulation of cytokine secretion	0.000746	CRP, APOA1, APOA2, IL1RAP, SRGN, SAA1, CD14
GO:0051224	negative regulation of protein transport	0.00081	APOA1, APOA2, APOD, SRGN, ADIPOQ
GO:1904950	negative regulation of establishment of protein localization	0.00081	APOA1, APOA2, APOD, SRGN, ADIPOQ
Molecular Function			
GO:0005319	lipid transporter activity	0.00279	APOF, APOA1, APOA2, APOD, APOM, RBP4
GO:0005496	steroid binding	0.00279	GC, APOF, APOA1, APOA2, APOD, SHBG
GO:0071813	lipoprotein particle binding	0.0101	CRP, APOA1, APOA2, THBS1
GO:0071814	protein-lipid complex binding	0.0101	CRP, APOA1, APOA2, THBS1
GO:0043178	alcohol binding	0.0105	APOF, APOA1, APOA2, APOD, RBP4
GO:0008289	lipid binding	0.0122	F10, GC, APOF, APOA1, APOA2, APOD, APOM, RBP4, SHBG, THBS1, CD14
GO:0005215	transporter activity	0.0128	GC, HBB, APOF, APOA1, APOA2, APOD, APOM, RBP4
GO:0022892	substrate-specific transporter activity	0.0170	HBB, APOF, APOA1, APOA2, APOD, APOM, RBP4
GO:0015485	cholesterol binding	0.0206	APOF, APOA1, APOA2, APOD
GO:0032934	sterol binding	0.0206	APOF, APOA1, APOA2, APOD

**Table 3:** Enrichment analysis of Gene ontology biological process and molecular function for differentially abundant proteins at T2, using WebGestalt online tool.

Gen set	Description	P-value	Overlap Gen-ID
Biological Process			
GO:0010976	positive regulation of neuron projection development	0.000151	FN1, IL1RAP, NRP1
GO:0045666	positive regulation of neuron differentiation	0.000151	FN1, IL1RAP, NRP1
GO:0050769	positive regulation of neurogenesis	0.000151	FN1, IL1RAP, NRP1
GO:0051962	positive regulation of nervous system development	0.000151	FN1, IL1RAP, NRP1
GO:0010975	regulation of neuron projection development	0.000262	FN1, IL1RAP, NRP1
GO:0031346	positive regulation of cell projection organization	0.000416	FN1, IL1RAP, NRP1
GO:0045664	regulation of neuron differentiation	0.000416	FN1, IL1RAP, NRP1
GO:0050767	regulation of neurogenesis	0.000618	FN1, IL1RAP, NRP1
GO:0051960	regulation of nervous system development	0.000618	FN1, IL1RAP, NRP1
GO:0031344	regulation of cell projection organization	0.00119	FN1, IL1RAP, NRP1
Molecular Function			
GO:0004888	transmembrane signaling receptor activity	0.0226	IL1RAP, NRP1
GO:0099600	transmembrane receptor activity	0.0226	IL1RAP, NRP1
GO:0004872	receptor activity	0.0304	PRG4, IL1RAP, NRP1
GO:0060089	molecular transducer activity	0.0304	PRG4, IL1RAP, NRP1
GO:0038023	signaling receptor activity	0.0336	IL1RAP, NRP1
GO:0004871	signal transducer activity	0.0533	IL1RAP, NRP1
GO:0016301	kinase activity	0.135	NRP1
GO:0016504	peptidase activator activity	0.135	FN1
GO:0016772	transferase activity, transferring phosphorus-containing groups	0.135	NRP1
GO:0016773	phosphotransferase activity, alcohol group as acceptor	0.135	NRP1

**Table 4:** Networks identified using IPA.

Top Diseases and Functions	Score	Focus Molecules	Molecules in Network
T1			
Lipid Metabolism, Molecular Transport, Small Molecule Biochemistry	45	19	Adipokine, ADIPOQ, APCS, APOA1, APOA2, APOA4, APOD, APOF, APOM, C1q, C4BP, C4BPA, Collagen type II, CRP, ERK1/2, F10, Fibrinogen, GC, GPLD1, Growth hormone, HDL, HDL-cholesterol, Histone h4, IL12 (complex), LDL, NADPH oxidase, Pro-inflammatory Cytokine, RARRES2, Rbp, RBP4, SAA1, SERPINF1, SERPINF2, Sod, TTR
Cardiovascular System Development and Function, Cellular Movement, Cell-To-Cell Signaling and Interaction	28	13	Akt, Ap1, collagen, Collagen Alpha1, Collagen type I, Collagen type IV, Collagen type VI, Collagen(s), DBH, DSG2, elastase, Fc gamma receptor, GP1BA, Gp2b3a Receptor, IL1, IL-1R, IL1RAP, Integrin, Iti, ITIH2, ITIH3, Laminin (complex), LCP1, Mek, Mmp, MMP9, p85 (pik3r), Pdgf (complex), PDGF BB, PEPD, SPARC, SRGN, Tgf beta, THBS1, VWF
Cellular Assembly and Organization, Nervous System Development and Function, Cell-To-Cell Signaling and Interaction	18	9	CD14, CFP, Cg, Creb, CRTAC1, ERK, estrogen receptor, F7, FAH, GOT, HBB, HISTONE, Histone h3, Ifn, IFN Beta, Ige, IgG, IgG1, IL12 (family), Immunoglobulin, Insulin, Mapk, NFkB (complex), NRP1, P38 MAPK, PI3K (complex), Pka, PRG4, Rac, RNA polymerase II, SHBG, SRC (family), TCF, Tnf (family), Vegf
Cell-To-Cell Signaling and Interaction, Hematological System Development and Function, Immune Cell Trafficking	15	8	ACTG2, Actin, APCS, APP, C7, CASP8, caspase, CCL17, CD3, CFI, CLEC10A, CNDP1, CTF1, CYTIP, Ddx3x, DEFA6, Dstn/Dstnl1, FETUB, Granzyme, HBG1, IL13, IL10RB, Jnk, LIPA, LRP1, MMRN1, MT1G, PZP, Ras, S100A12, SCAVENGER receptor CLASS A, Sos, SRGN, TCR, ZFP36L1
Inflammatory Response, Infectious Diseases, Inflammatory Disease	5	3	ADIPOR2, APCS, BBS12, CD163, CD276, CLEC10A, CMKLR1, coagulation factor, CYP19, CYTIP, estrone, FBXO42, Fcgr2, FSH, glycosylphosphatidylinositol, GPER1, GPR119, HFE, IL6, IL17B, IL1RL2, LPAR2, N-cor, NfkB1 RelA, PAX8, Proinsulin, RARRES2, S100A12, SCAVENGER receptor CLASS A, SCN9A, SELENOS, SHBG, SMPDL3A, Timp, TNF
**T2**			
Cell-To-Cell Signaling and Interaction, Cell Death and Survival, Inflammatory Response	21	7	Akt, ANGPTL1, B4GALT5, C4BPB, CHST3, CLEC10A, Collagen type VI, CRP, Enolase, ERK, ERK1/2, EXT1, Fc gamma receptor, Fc receptor, FCER1A, FCN1, FN1, GFRA2, HS6ST1, IL1, IL6, IL1RAP, ITIH3, miR-191-5p (and other miRNAs w/seed AACGGAA), NFkB (complex), NfkB1-RelA, NRP1, PRG4, PRSS38, pyridoxamine, S100A12, SEMA3D, SHBG, Stat3-Stat3, TGFB1

## 2 Experimental design, materials and methods

### 2.1 Study cohort and sample treatment

A total of 16 obese subjects undergoing LGP was investigated at three timepoints; pre-surgery (n = 16), at 1–2 months post-surgery (T1, n = 16), at 4–5 months post-surgery (T2, n = 9). The detailed characteristics of patients were mentioned in Savedoroudi et al. [1]. The six most abundant serum proteins (albumin, IgG, IgA, antitrypsin, transferrin and haptoglobin) were depleted in the serum samples using the Agilent Multiple Affinity Removal column (4.6 × 50 mm) according to the instructions recommended by the manufacturers (Agilent Technologies, CA, USA). Then, the filter-aided sample preparation (FASP) protocol was utilized to prepare samples as described previously [1], [3]. Written consent was obtained from all participants and the institutional review board and the ethic committee of Shahid Beheshti University approved the study protocol for Human rights (IR.SBU.ICBS.97/1019).

### 2.2 LC-MS/MS analysis

Initially, the peptides were resuspended in 2% acetonitrile, 0.1% FA and 0.1% trifluoroacetic acid. LC-MS/MS analysis was carried out on a UPLC-nanoESI MS/MS setup with a Dionex RSLC nanopump (Dionex, CA, USA). The system was coupled online with an emitter for nanospray ionization (New objective picotip 360-20-10) to a Q Exactive HF mass spectrometer (Thermo Scientific, Waltham, USA). The samples were analyzed in a random order, in triplicates. The peptide material was loaded onto a 2cm C18 trapping column (Dionex Acclaim PepMap RSLC C18) and separated using a 75cm C18 reversed-phase column (Dionex Acclaim PepMap RSLC C18). Both columns were kept at 60 °C. The peptides were eluted with a gradient of 98% solvent A (0.1% FA) and 2% solvent B (0.1% FA in acetonitrile), which was increased to 8% solvent B on a 5-min ramp gradient and subsequently to 30% solvent B on a 45-min ramp gradient, at a constant flow rate of 300nL/min. The mass spectrometer was operated in positive mode using a Top15 data-dependent MS/MS scan method. A full MS scan in the 375–1500 m/z range was acquired at a resolution of 120 000 with an AGC target of 3 × 10^6^ and a maximum injection time of 50 ms. Fragmentation of precursor ions was performed by higher-energy C-trap dissociation with a normalized collision energy of 27. MS/MS scans were acquired at a resolution of 15000 with an AGC target of 2 × 10^5^; maximum injection time was 100 ms. Dynamic exclusion was set to 5s.

### 2.3 Data analysis and processing

Mass spectrometry data were analyzed in MaxQuant version 1.6.0.1 and searched against the Uniprot human reference FASTA database (August 2017) [4], [5]. Label-free protein quantitation (LFQ) algorithm was performed with a minimum ratio count of 1. Standard settings in MaxQuant were employed, including carbamidomethylation of cysteines as a fixed modification, and acetylation of protein N-terminals, oxidation of methionine, and deamidation of asparagine and glutamine as variable modifications. A maximum of two tryptic missed cleavages was allowed. The false discovery rate (FDR) of identified proteins and peptides was set to a maximum of 1%, using a target-decoy fragment spectra search strategy. Hereby, high confidence identifications were ensured. The “match between runs” feature was enabled to transfer peptide identifications across LC-MS/MS runs, based on accurate retention time and mass-to-charge. The output from MaxQuant, containing the list of proteins identified below 1% FDR, was further filtered and processed in Perseus version 1.6.0.2 [6]. All reverse hits and proteins identified only by site were removed from further analysis, and the data were log 2-transformed. At least two unique peptides were required for a protein quantitation. Additionally, the unique peptides were required to be quantifiable in at least 70% of samples.

### 2.4 Bioinformatics analysis

Differentially regulated proteins between pre- and post-surgery subjects were functionally categorized based on gene ontology (GO) classification using WEB-based GEne SeT AnaLysis Toolkit (WebGestalt) [7]. Identification of networks was performed with Ingenuity Pathway Analysis software (IPA; Ingenuity Systems, Redwood City, CA, www.ingenuity.com). Gene symbols and the corresponding protein fold change were imported to IPA software using core analysis. Standard settings in IPA were employed, including: direct and indirect relationships between focused molecules with default settings of 35 molecules/network, based on experimentally observed data (high confidence predictions and moderate confidence interactions excluded) were considered. All sources of data from human, mouse and rat studies in the Ingenuity Knowledge Base were included.
